# Changes in community composition of ammonia-oxidizing betaproteobacteria from stands of Black mangrove (*Avicennia germinans*) in response to ammonia enrichment and more oxic conditions

**DOI:** 10.3389/fmicb.2013.00343

**Published:** 2013-11-20

**Authors:** Hendrikus J. Laanbroek, Rosalinde M. Keijzer, Jos T. A. Verhoeven, Dennis F. Whigham

**Affiliations:** ^1^Department of Microbial Ecology, Netherlands Institute of Ecology (NIOO-KNAW)Wageningen, Netherlands; ^2^Ecology and Biodiversity Group, Institute of Environmental Biology, Utrecht UniversityUtrecht, Netherlands; ^3^Smithsonian Environmental Research CenterEdgewater, MD, USA

**Keywords:** ammonia oxidation, betaproteobacteria, mangroves, slurries, oxygen

## Abstract

In flooded and non-flooded impounded forests of Black mangrove (*Avicennia germinans*), the community structure of the ammonia-oxidizing betaproteobacteria (β-AOB) differed among distinct mangrove vegetation cover types and hydrological regimes. This had been explained by a differential response of lineages of β-AOB to the prevailing soil conditions that included increased levels of moisture and ammonium. To test this hypothesis, slurries of soils collected from a flooded and a non-flooded impoundment were subjected to enhanced levels of ammonium in the absence and presence of additional shaking. After a period of 6 days, the community composition of the β-AOB based on the 16S rRNA gene was determined and compared with the original community structures. Regardless of the incubation conditions and the origin of the samples, sequences belonging to the *Nitrosomonas aestuarii* lineage became increasingly dominant, whereas the number of sequences of the lineages of *Nitrosospira* (i.e., Cluster 1) and *Nitrosomonas* sp. Nm143 declined. Changes in community structure were related to changes in community sizes determined by quantitative PCR based on the *amoA* gene. The *amoA* gene copy numbers of β-AOB were compared to those of the ammonia-oxidizing archaea (AOA). Gene copy numbers of the bacteria increased irrespective of incubation conditions, but the numbers of archaea declined in the continuously shaken cultures. This observation is discussed in relation to the distribution of the β-AOB lineages in the impounded Black mangrove forests.

## Introduction

Aerobic ammonia-oxidizing archaea and bacteria play an important role in the global nitrogen cycle by converting ammonia to nitrite (Kowalchuk and Stephen, 2001; Schleper and Nicol, 2010). Nitrite can then be further oxidized to nitrate by nitrite-oxidizing bacteria or used as an electron acceptor in a large number of microbial and chemical redox reactions. Studies applying the phylogenetic 16S rRNA gene and the *amoA* gene that codes for the α-subunit of the enzyme ammonia monooxygenase in aerobic ammonia-oxidizing archaea and bacteria showed that the ammonia-oxidizing thaumarchaea and betaproteobacteria (β-AOB) are widely distributed (Kowalchuk and Stephen, 2001; Schleper and Nicol, 2010) and occur in many habitats, including soils of mangrove forests (e.g., Wickramasinghe et al., 2009.) Mangroves are tree-dominated intertidal wetlands along tropical and subtropical coastlines with a specialized flora adapted to waterlogged and saline conditions.

In a previous study (Laanbroek et al., 2012) we investigated the effects of vegetation cover type and summer flooding on the process of ammonia oxidation in soils collected from mangrove stands that were dominated by Black mangrove (*Avicennia germinans*) of differing statures that occurred in impoundments with dissimilar hydrologic regimes. The vegetation cover type did not significantly affect the potential ammonia-oxidizing activity (PAA) and a possible effect of summer flooding was offset by the effect of an extremely dry winter yielding high pore water salinities. In the same study (Laanbroek et al., 2012) we investigated also the distribution of different lineages of ammonia-oxidizing betaproteobacteria (β-AOB) in soils from different mangrove vegetation cover types. Based on the 16S rRNA gene, the majority of the β-AOB in soils from these impoundments was related to the lineages of *Nitrosomonas aestuarii/Nitrosomonas marina*, *Nitrosospira* (Cluster 1) and *Nitrosomonas* Nm143, irrespective of the hydrologic regime. Sequences related to the *N. aestuarii/N. marina* lineage were mostly encountered at drier locations in the impoundments. In contrast, sequences associated with the *Nitrosomonas* Nm143 lineage were mostly observed at the wetter sites. Since higher moisture levels are generally associated with more reduced conditions in the soils, it can be argued that members of the *Nitrosomonas* Nm143 lineage are better adapted to such reduced conditions. Sequences related to *Nitrosospira* Cluster 1 were mostly found at places with low nutrient availability as had been observed before in a freshwater marsh (Laanbroek and Speksnijder, 2008). Hence, the lineages of β-AOB were differently affected by a number of environmental factors.

To test the hypothesis that ammonium availability and redox conditions impacted the characteristics of the β-AOB community in the soils dominated by Black mangrove, changes in the composition of the communities were determined in ammonium-enriched, shaken, or non-shaken soil suspensions that were incubated for 6 days at room temperature. It was assumed that shaking resulted in more oxic conditions for the aerobic ammonia-oxidizing cells. Coci et al. (2005) had previously shown that the species composition of β-AOB communities can respond quickly to changes in environmental conditions and that oxic conditions are a prerequisite for changes in the community structure. Based on their distribution in the Black mangrove soils, we expected that gene numbers of *Nitrosomonas* lineages would increase in the nutrient-enriched soil suspensions at the cost of *Nitrosospira* Cluster 1, irrespective of shaken or non-shaken conditions, and that shaking of the suspensions will give the *N. aestuarii/N. marina* lineage extra advantage over the *Nitrosomonas* Nm143 lineage. Changes in community structure of the β-AOB due to 6 days prolonged incubation were related to changes in community sizes determined by quantitative PCR based on the *amoA* gene. The *amoA* gene copy numbers of β-AOB were compared to those of the ammonia-oxidizing archaea (AOA).

## Materials and methods

### Study locations

The two mangrove-dominated impoundments that we studied, and described in detail in Laanbroek et al. (2012), are located in the Indian River Lagoon, a coastal sub-estuary, located on North Hutchinson Island, St. Lucie County, Florida, USA. For the experiments and analyses described in the present paper, samples were collected in March 2009 and March 2010 just after a new management regime of spring and summer flooding was initiated for purposes of controlling the numbers of salt-marsh mosquitoes and biting midges in one of the impoundments (Impoundment 24). Hence, soils collected in March 2009 and March 2010 experienced different hydrologic regimes in Impoundment 24 during the preceding spring and summer period resulting in increased days of total soil submergence (Verhoeven et al., under review). For comparison, soil samples were also collected from the adjacent Impoundment 23 that was not subjected to the new management. Soil samples used in this study were collected from three different vegetation cover types, each dominated by Black mangrove (*Avicennia germinans*). The three cover types (dwarf, sparse, and dense) are described in Laanbroek et al. (2012) and Verhoeven et al. (under review). Within each impoundment, soils from five locations in each of the three cover types were collected resulting in a total of 30 locations sampled.

### Soil collections

In March 2009 and March 2010, soil cores (3.9 cm diameter and 10 cm long) were collected at each of the 30 sites. Samples were collected with an aluminum tube that was sharpened at one end. The cores were immediately sealed at both ends with rubber stoppers and transported to the laboratory. One of the cores was used for removal of pore water that was analyzed for salinity and pH. The top 5-cm of two other cores were combined and thoroughly mixed by hand and subsequently sub-sampled for the determination of bulk density and moisture content, for measurements of potential rates of nitrification and for the analysis of the community structure of β-AOB. Samples for the genetic analyses were freeze-dried immediately after mixing of the sampling cores and stored until further processing.

### Slurry experiments

PAAs were determined in slurries of 20 g fresh weight soil mixed with 50 ml of mineral medium containing ammonium at a final concentration of 1 mM (see Laanbroek et al., 2012 for details). Central to this PAA method is the determination of the accumulation rate of nitrite plus nitrate being together the sum of ammonia oxidized by the microorganisms present in the soil samples. After the initial measurements of 9 h under optimal conditions (i.e., shaken) at room temperature, the slurries were left at room temperature for 6 days, either non-shaken (2009 samples) or shaken (2010 samples). At day 6, the PAA measurements were repeated after 1mM ammonium had again been added, while all slurries were aerated again; hence, both in 2009 and 2010. After the last sampling on day 6, soil particles in the slurries that had settled out for 1 h were removed and subsequently freeze-dried for genetic analyses. In total, 30 slurry samples were measured each year, i.e., slurries from quintuplet soil samples from three different vegetation cover types in two different impoundments.

### DNA isolation

DNA isolation, PCR, and construction of clone libraries were similar to the procedures described for the freshly collected soil samples as described before (Laanbroek et al., 2012). From each of the slurry samples that were freeze-dried after the last measurement on day 6, 0.5 g was homogenized in 1 ml cetyltrimethylammonium bromide (CTAB) buffer (Zhou et al., 1996) in the presence of 0.5 g sterilized zirconia-silica beads (diameter 0.1 mm), subjected to disruption by bead-beating at a 5.0-m/s rotation for 60 s and subsequently incubated for 30 min at 37°C in the presence of 5 μl proteinase K (20 mg/ml). After vortexing for 15 min, the samples were supplemented with 150 μl of a 20% SDS solution and incubated for 1 h at 65°C in a Thermoblock and again vortexed every 15–20 min. After centrifugation at 10,000 × *g* for 10 min, 600 μl of supernatant was collected in 2-ml screw-cap tubes. The rest of the sample was re-extracted with 450 μl CTAB buffer and 50 μl of a 20% SDS solution, vortexed for 10 s, incubated for 10 min at 65°C, and centrifuged at 6,000 × *g* for 10 min. Again, 600 μl was collected, added to the previously extracted supernatant, mixed with 1 ml phenol-chloroform-isoamyl alcohol solution (25:24:1, vol/vol/vol), and centrifuged at 6,000 × *g* for 10 min. One milliliter of supernatant was collected and placed into a new screw-cup tube containing 700 μl isopropanol, and the tube was incubated for 1 h at 24°C. After 20 min of centrifugation at 15,000 × *g*, the isopropanol was decanted and the pellet was resuspended and washed with 1 ml 70% cold ethanol. This was followed by 5 min of centrifugation at 15,000 × *g*, decantation of the ethanol, drying of the pellet under vacuum centrifugation, and finally, resuspension in 100 μl water (Sigma).

### PCR and construction of clone libraries based on the 16s rRNA gene

Extracted DNA was amplified by nested procedure using two 16s rRNA gene primer sets specific for the majority of the betaproteobacterial AOB, i.e., the βAMO161f and βAMO1301r primer set of McCaig et al. (1994) and the CTO189f and CTO654r primer set of Kowalchuk et al. (1997). One hundred ng of purified DNA was used as template for a 50-μl PCR mixture containing 1 × Mg-free buffer (Invitrogen Corp., Carlsbad, CA), 0.5 μM of each primer, 200 μM of each deoxynucleotide triphosphate, 1.75 mM MgCl_2_, 400 ng/μl bovine serum albumin, 1.25 U GoTaq Hot Start Polymerase (Promega). The thermocycling program for both steps consisted of 2 min of denaturation at 95°C followed by 35 cycles of 30 s of denaturation at 95°C; 30 s of specific annealing at 59°C (βAMO primer set) or at 57°C (CTO primer set) and 45 s of elongation at 72°C; 5 min of final elongation was performed for all reactions. Nested amplifications of 25 cycles were performed with the primer set CTO189f and CTO654r on 1:100 dilutions of PCR products from the βAMO primer set. All reactions were verified by UV illumination of 1% agarose gels stained in a gel red or ethidium bromide solution. Polymerase chain reaction fragments were ligated into the pGEM T-vector system (Promega Corporation, Madison, WI, USA) and transformed into JM109 competent *E. coli* cells (Promega) according to the manufacturers' instructions. Transformed colonies were screened for inserts of the correct size by PCR. Sanger sequencing using primer T7 was performed by Macrogen, Amsterdam, Netherlands.

### Sequence analyses

Sequences were aligned and checked for chimeras using Sequencher 4.1 (Gene Codes Corporation, Ann Arbor, MI). The chromatogrammes of the remaining sequences were visually inspected and wrongly assigned bases were manually replaced. Low quality sequences were removed from the dataset. After removing sequences of insufficient length or quality, the remaining 1022 sequences were further analyzed. The aligned sequences were clustered in Operational Taxonomic Units (OTUs) with the Cluster program of MOTHUR software version 1.18.1 (Schloss et al., 2009). In addition, MOTHUR was also used for establishing rarefaction curves and group (i.e., OTU-) representatives. OTUs at the level of 97% mutual similarity in their 16S rRNA gene sequences (418 bp) were chosen for further analyses. Sequence identification of the OTU-representatives was done by the BLASTN facility from the National Center for Biotechnology Information. (http://www.ncbi.nlm.nih.gov/). The partial 16SrRNA gene sequences were submitted to the GenBank database under accession numbers JQ725556—JQ726359 (fresh soils) and KF719449—KF719951 (incubated slurries).

Analysis of similarity (ANOSIM) between communities of β-AOB obtained from different impoundments, years, mangrove cover types and incubations times of the slurries was done with the PRIMER software version 5.2.9 (Primer-E, Plymouth, UK). The ANOSIM analysis was based on Bray-Curtis distance matrices.

### Quantitative PCR of archaeal and bacterial *amoA* genes

Gene copy numbers of archaeal and bacterial *amoA* genes were determined in separate reactions with the primer sets ArchamoA-1F and ArchamoA-2R (Francis et al., 2005) and amoA-1F and amoA-2R (Rotthauwe et al., 1997), respectively. The reaction mixture consisted of 10 μL Rotor-Gene SYBR Green PCR kit (Qiagen), 1 μL of each primer (5 pmol μL^−1^), 10 μg bovine serum albumin (100 mg ml^−1^, New England Biolabs Inc.) and 5 μL template DNA (approximately 50 ng total soil DNA); final volume was 20 μl. The PCR conditions for the archaeal *amoA* gene were 15 min at 95°C, followed by 40 cycles of 45 s at 95°C, 45 s at 55°C and 45 s at 72°C, and finally 5 min at 72°C. The PCR conditions for the bacterial *amoA* gene were 5 min at 95°C, followed by 45 cycles of 45 s at 95°C, 45 s at 58°C and 45 s at 72°C, and finally 5 min at 72°C. Standard curves for quantification of bacterial and archaeal *amoA* genes were generated from10-fold serial dilutions (10^2^–10^8^ copies μL^−1^) of plasmid DNA containing either a bacterial *amoA* fragment of 635bp that was obtained from a pure culture of *Nitrosomonas europaea* ATCC 19718 or an archaeal *amoA* fragment from clone 29C_47 [accession number JQ404089, (Daebeler et al., 2012)]. The detection limits for the qPCR assay were 2.83 × 10^3^ and 9.81 × 10^2^ gene copies/g of dry soil for the archaeal and bacterial *amoA* genes, respectively, which corresponds to three copies per reaction. qPCR was performed in a Corbett Research Rotorgene 3000 Cycler, (Qiagen, Netherlands).

### Statistical analyses

In contrast to the log-transformed bacterial *amoA* gene copy numbers, the log-transformed archaeal *amoA* gene copy numbers were not normally distributed. Therefore, nonparametric Spearman rank order correlation and Kruskal-Wallis analysis of variation were applied for both archaeal and bacterial *amoA* gene copy numbers. The statistical analyses were performed with the STATISTICA software version 12 (StatSoft Inc., Tulsa, OK).

## Results

### Potential ammonia-oxidizing activities

**Figure 1:**
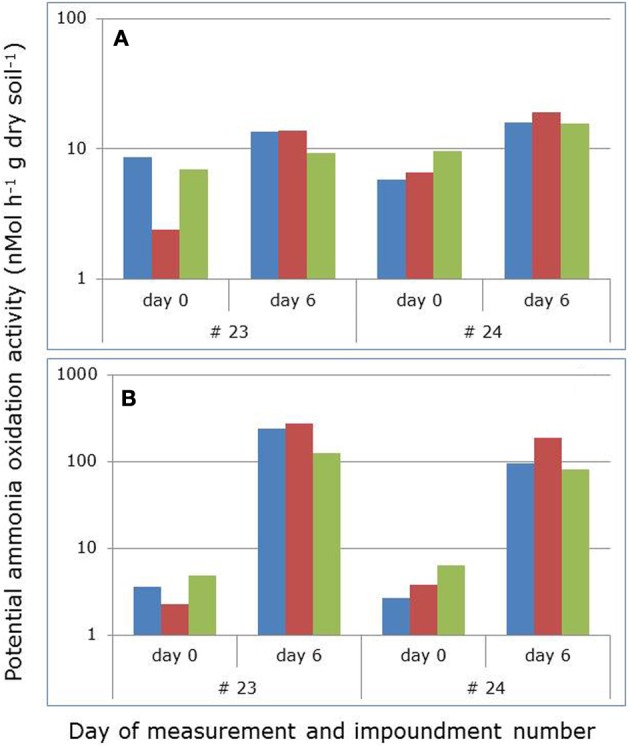
**Average potential ammonia-oxidizing activities in mangrove soil slurries before and after prolonged incubation of 6 days with added ammonium in the absence (A) and presence (B) of continuously shaking of the slurries.** Soils had been collected in 2009 and 2010 in two different impoundments (23 and 24) from three different habitats characterized by stunted (blue), sparse (red), and dense (green) *Avicennia germinans*. Both in 2009 and in 2010, incubation increased the potential activities significantly (*p* = 0.0039 and *p* = 0.0000, respectively; non-parametric Kruskal-Wallis test with *N* = 60 and *H* = 8.344 and *H* = 27.876, respectively). No significant effects between years, impoundments, and vegetation cover types were observed.

PAAs in the soil slurries increased significantly (*p* = 0.0039 and *p* < 0.0001) in 2009 and 2010 with prolonged incubation for 6 days in the laboratory (Figure 1). The increase in activity was most pronounced in 2010 when shaking of the ammonium-enriched slurries continued during the whole incubation period. In 2009, shaking had been discontinued between the potential activity measurements on day 0 and 6.

### OTUs of β-AOB present in mangrove soil samples

Based on 97% mutual similarity between the 16S rRNA genes (418 bp), the 1067 qualified sequences could be classified into 35 different OTUs. Of these 35 OTUs, 17 contained only a singleton, and 10 others comprised only 2–4 sequences. The remaining eight OTU's contained at least eight sequences and were used for the analyses of community composition. These eight OTUs comprised 320 and 220 sequences obtained from the original mangrove soils in 2009 and 2010, respectively, and 221 and 261 sequences from the incubated slurries at the end of the incubation experiments in 2009 and 2010, respectively. A BLAST analysis of the eight OTU's representatives yielded a limited number of lineages of β-AOB (Table 1). Except for OTU05, which sequences were 100% similar to a sequence obtained from a nitrifying bioreactor, all other OTUs contained sequences that were mostly related to sequences originating from estuarine, marine or saline environments.

**Table 1:** **BLAST analysis of OTU's representatives encountered in Black mangrove soils collected from the Indian River lagoon at North Hutchinson Island near Fort Pierce, Florida**.

OTU #	**BLAST analysis of OTU's representatives**			
	**Closest type strain**	**% similarity**	**Origin closest relative**	**References**
01	*Nitrosomonas aestuarii*	97	High altitude saline wetland	Dorador et al., 2008
02	*Nitrosospira tenuis*	96	Deep water sponge	Meyer and Kuever, 2008
03	*Nitrosomonas* sp. Nm143	97	Estuarine sediment	Jin et al., 2011
04	*Nitrosomonas aestuarii*	98	Prawn farm sediment	Ma and Wang, 2007
05	*Nitrosomonas europaea*	100	Nitrifying bioreactor	Bae and Song, 2011
06	*Nitrosomonas aestuarii*	97	Estuarine sediment	Magalhaes et al., 2007
07	*Nitrosomonas aestuarii*	98	Estuarine sediment	Freitag et al., 2006
08	*Nitrosomonas aestuarii*	96	Coastal marine sediment	Urakawa et al., 2006

Only OTUs with numbers of sequences larger than 1% of the total number of sequences.

### Incubation-induced changes in community composition in slurries

**Figure 2:**
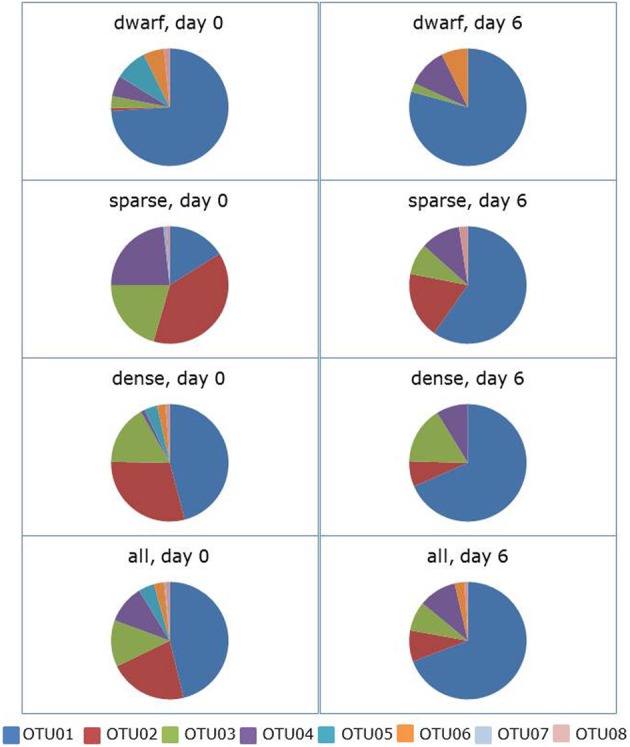
**Effect of 6 days of ammonium enrichment on the sequence distribution of ammonia-oxidizing betaproteobacteria in non-shaken soil slurries.** Sequences originated from different vegetation cover types of *Avicennia germinans* forests sampled in 2009. Operational Taxonomic Units (OTUs) are based on the partial 16S rRNA gene (440 bp).

Incubation of the soil slurries in the absence of shaking led to a significant change in community composition (ANOSIM: *R* = 0.084, *p* = 0.050). The average dissimilarity between the communities at the start and the end of the incubation period was 59%. OTU01 contributed most (i.e., 39%) to this dissimilarity followed by OTU02 and OTU04 with 24 and 15%, respectively (Figure 2, supplementary Table 1). The relative presence of sequences belonging to OTU01 (related to *N. aestuarii*) increased from 46 to 69%. With an increase of 6–22% of the total sequences, the increase in OTU01 was most pronounced in the samples from locations with the sparse vegetation cover types. In the same period of 6 days, the relative occurrence of sequences belonging to OTU02 (related to *Nitrosospira* Cluster 1) decreased from 22 to 9% of the total number of sequences. This relative decrease in presence was observed for all vegetation cover types. The relative changes in occurrence of sequences belonging to the other OTUs were small except that sequences belonging to OTU05 (comprising the *N. europaea* lineage) and OTU07 (consisting of sequences mostly related to the *N. aestuarii/N. marina* lineage) that disappeared completely from the clone libraries.

**Figure 3:**
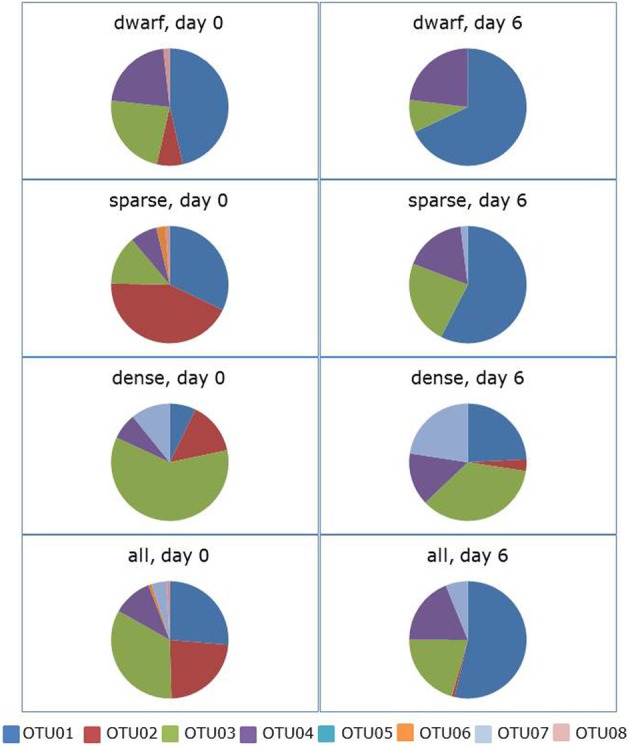
**Effect of 6 days of ammonium enrichment on the sequence distribution of ammonia-oxidizing betaproteobacteria in shaken soil slurries.** Sequences originated from different vegetation cover types of *Avicennia germinans* forests sampled in 2010. Operational Taxonomic Units (OTUs) are based on the partial 16S rRNA gene (440 bp).

Incubation of the soil slurries in the presence of shaking led also to a significant change in community composition (ANOSIM: *R* = 0.149, *p* = 0.014).The average dissimilarity between the communities at the start and the end of the incubation period was 64%. OTU01 contributed again most (i.e., 35%) to this dissimilarity followed this time by OTU03 and OTU02 with 24 and 17%, respectively (Figure 3, Supplementary Table 2). The relative presence of sequences belonging to OTU01 more than doubled from 26 to 54% of the total sequences. This was true for all three vegetation cover types, except for the dwarf vegetation type where OTU01 comprised already almost half of the sequences before the incubation started. Sequences belonging to OTU02 almost disappeared from the clone libraries with only a few sequences left in the dense vegetation cover type. With a decrease from 23 to 8% of the total, the relative occurrence of sequences belonging to OTU03 declined especially in the soil slurries originating from the dense vegetation cover type. The relative changes in occurrence of sequences belonging to the other OTUs were small. However, the few sequences belonging to OTU02 and OTU08 disappeared completely from the slurries.

### *amoA* gene copy numbers before and after prolonged incubation

**Figure 4:**
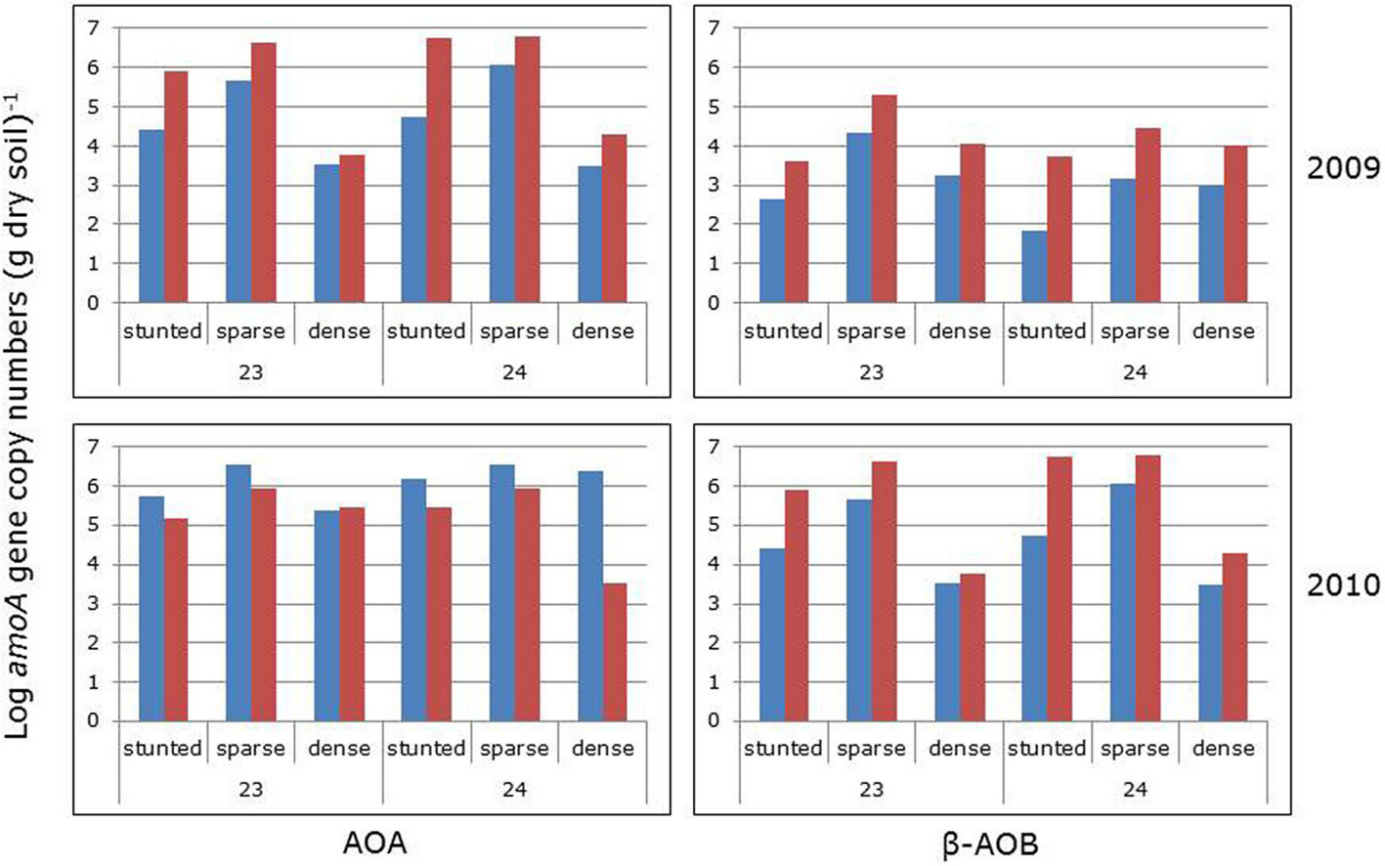
**Archaeal (left panels) and bacterial (right panels) *amoA* gene copy numbers (numbers per g dry soil) before (blue bars) and after (red bars) prolonged incubation of 6 days with added ammonium in the absence (2009 series) and presence (2010 series) of continuously shaking of the slurries.** Soils had been collected in two different impoundments (23 and 24) from three different habitats characterized by stunted, sparse, and dense *Avicennia germinans*. For statistical data see Table 2.

Averaged for the sampling groups, numbers of archaeal *amoA* genes were always larger than the numbers of bacterial *amoA* genes, irrespective of the year of sampling, the impoundment from which the samples were collected and the mangrove vegetation cover type (Figure 4). For individual samples, the ratios between archaeal and bacterial gene copy numbers varied largely with the lowest (i.e., 0.9) and highest (6.5 × 10^4^) ratios found in 2009 in the sparse vegetation of impoundment 23 and impoundment 24, respectively. Gene copy numbers of both AOA and β-AOB were significantly larger in 2010 compared to 2009 (Table 2). The impoundment from which the soil samples had been collected had no significant effect on the gene copy numbers, although the ratio of archaeal to bacterial gene copy numbers were significantly higher in impoundment 24. On average, both archaeal and bacterial gene copy numbers were larger in the sparse mangrove vegetation compared to the other cover types; however, there was no significant difference in the bacterial gene copy numbers between the sparse and the dense mangrove vegetation types.

**Table 2:** **Effect of year of sampling, impoundment and type of Black mangrove vegetation on the archaeal and bacterial amoA gene copy numbers (AOA and AOB, respectively) and their mutual ratio according to a nonparametric Kruskal-Wallis test**.

		***N***	**Degrees of freedom**	***p***	**Remarks**
Year	log AOA	55	1	0.0014	2010 > 2009
	log AOB	55	1	0.0959	2010 > 2009
	log AOA/AOB	52	1	0.6905	
Impoundment	log AOA	55	1	0.7738	
	log AOB	55	1	0.1340	
	log AOA/AOB	52	1	0.0197	24 > 23
Vegetation type	log AOA	55	2	0.0067	SP > ST = DE^1^
	log AOB	55	2	0.0024	SP = DE > ST
	log AOA/AOB	52	2	0.0020	SP = DE > ST

1 ST, stunted; SP, sparse; DE, dense.

The archaeal and bacterial gene copy numbers were significantly correlated with each other (Supplementary Table 3: Spearman rank order correlation). Both correlated also significantly with extractable soil phosphate (positively) and with pore water pH and nitrate (negatively). The archaeal *amoA* gene copy numbers, but not the numbers of β-AOB was significantly and negatively correlated with pore water salinity. Finally, neither archaeal nor bacterial *amoA* gene copy numbers correlated with the PAA.

Prolonged incubation for 6 days in the presence of added ammonium increased both the archaeal and the bacterial *amoA* gene copy numbers (Figure 4; 2009 data series). These increases were significant (Table 3). However, the copy numbers of the β-AOB increased more leading to a significantly lower AOA to β-AOB ratio. Prolonged incubation for 6 days in the presence of both added ammonium and shaking had a significant negative effect on the archaeal *amoA* gene copy numbers, whereas the numbers of the bacterial *amoA* genes increased (Figure 4; 2010 data series). Again, the ratio between archaeal and bacterial gene copy numbers declined significantly during the period of prolonged incubation.

**Table 3:** **Effect of incubation on the numbers of ammonia-oxidizing archaea and bacteria and their mutual ratio according to a nonparametric Kruskal-Wallis test**.

	**Year of sampling**	***N***	**Degrees of freedom**	***H***	***p***	**Remarks**
log AOA	2009	55	1	7.044	0.0080	day 6 > day 0
	2010	59	1	15.603	0.0001	day 6 < day 0
log AOB	2009	53	1	9.284	0.0023	day 6 > day 0
	2010	59	1	0.568	0.4439	day 6 > day 0
log AOA/AOB	2009	50	1	2.992	0.0837	day 6 < day 0
	2010	58	1	5.126	0.0236	day 6 < day 0

## Discussion

The hypothesis that enrichment with ammonium and more oxic conditions would favor members of the *N. aestuarii/N. marina* lineage more than the other lineages present in the soil samples was confirmed by the slurry experiments. During the slurry experiments, OTU01 related to the *N. aestuarii/N. marina* lineage, which was dominant in the original soil samples (Figures 1, 2) became even more dominant at the cost of OTU02 belonging to *Nitrosospira* Cluster 1 and to a lesser extent at the expense of OTU03, which is related to the *Nitrosomonas* sp. Nm143 lineage. The changes observed in ammonium-enriched, non-shaken soil slurries were amplified strongly in ammonium-enriched, shaken incubations. The more oxic conditions of the soil suspensions due to shaking strengthened the effect of incubation on the relative presence of the different lineages compared to the treatment without shaking. Apparently, the presence of both ammonium and more oxic conditions offered members of the *N. aestuarii/N. marina* lineage conditions for growth that enabled them to overgrow their competitors. The large decline with 15% in the relative occurrence of sequences belonging to OTU03 when exposed to shaking might reflect the adaptation of the *Nitrosomonas* sp. Nm143 to more reduced conditions as these likely prevail in the moist dense vegetation cover type in the impoundments (Verhoeven et al., under review). Such a large decline in the relative occurrence of sequences belonging to OTU03 was not observed in the non-shaken soil slurries. During incubation of the soil slurries, the relative decline in the number of sequences related to the *Nitrosomonas* sp. Nm143 lineage was smaller than the decline in the number of sequences belonging to *Nitrosospira* Cluster 1. This could be due to a yet unknown characteristic that enables *Nitrosomonas* sp. Nm143 to counterbalance the dominant *N. aestuarii/N. marina* lineage better or for a longer period than the *Nitrosospira* lineage.

Incubation of the slurries resulted in an increase in the PAAand in the community composition of β-AOB over a period of 6 days. The increased activity was apparently related to the observed increases in bacterial *amoA* gene copy numbers in both years. Similar to the bacterial gene numbers, the archaeal *amoA* gene copies also increased in numbers during the prolonged 6 days incubation period, but only when ammonium had been added in the absence of prolonged shaking. When the soil slurries were continuously shaken during the incubation period, archaeal gene copy numbers declined. Hence, AOA may be less resistant to more oxic conditions as caused by continuously shaking of the soil suspensions. Data on ratios between AOA and β-AOB gene copy numbers in mangrove forest soils are relative scarce and nothing is known about the effect of redox conditions on this ratio. The numbers of bacterial *amoA* genes in a *Kandelia obovata*-dominated mangrove forest in the Mai Po Nature Reserve in the New Territories of Hong Kong outnumbered those of archaeal *amoA* genes by a factor of 5–12 (Li et al., 2011). In mangrove soil microcosms from the same area, addition of addition of ammonium led to an increase in *amoA* gene copy numbers of both AOA and β-AOB, but the latter remained numerically dominant (Li and Gu, 2013). Studies with respect to the presence of ammonia-oxidizing gammaproteobacteria in mangrove forest soils are limited. Sequences belonging to this bacterial group could not be demonstrated in this habitat (Wickramasinghe et al., 2009).

Aerobic degradation of labile materials near the surface of mangrove sediments happens usually so fast that oxygen rarely penetrates more than 2 mm into the sediment (Kristensen et al., 1994). Redox potentials of *Avicennia*-dominated sediments at the coast of Queensland, Australia, varied from 0 till −200 mV at the surface and reached values from −100 till −300 mV at 5 cm depths (Clark et al., 1998). The *Avicennia*-dominated forest soils that we use in our incubation experiments had been exposed to atmospheric oxygen for several months prior to sampling. This was particularly true for the dwarf and sparse vegetation cover types. Nevertheless, it is likely that a part of the aerobic ammonia-oxidizing community in the upper 5 cm of the soil that had been collected was inactive at the time of sampling due to oxygen limitation.

What do the observations tell us about the distribution of lineages of β-AOB in the mangrove soils from which the samples were collected? Sequences belonging to the *N. aestuarii/N. marina* lineage were most numerous among the β-AOB encountered in the Black mangrove soils from the impoundments along the Indian River lagoon. They accounted for nearly 50 percent of the β-AOB community. Hence, it may be concluded that the conditions in the impoundments are especially suitable for the *N. aestuarii/N. marina* lineage. Next to the conditions indicated above, salinity may be an additional factor favoring the *N. aestuarii/N. marina* lineage. The share of sequences belonging to OTU01 was higher in 2009 compared to 2010 (Tables 2, 3), which might have been due to average higher salinities measured in 2009 compared to 2010. However, the slurry experiments were performed at their own field salinity, which means in ranges of 52–88% and 28–54% in 2009 and in 2010 respectively, which likely exclude salinity as factor that induced changes in the communities of β-AOB during slurry incubations.

The Black mangrove forests in the impoundments along the Indian River lagoon are rather atypical for this type of ecosystem in the sense that tide is largely reduced. In addition, Impoundment 24 had been submerged during the summer season of 2009, which resulted in higher ammonium concentrations in the pore water (Verhoeven et al., under review). Especially the dwarf and sparse black mangrove vegetation cover types have been exposed directly to the atmosphere for several months during the autumn and winter period before the samples were collected. Under these exposed conditions with increased ammonium concentrations, members of the *N. aestuarii*/*N. marina* lineage become apparently the dominant β-AOB.

The lineages of β-AOB as detected in the Black mangrove soils are commonly observed in estuaries (Francis et al., 2003; Bernhard et al., 2005, 2007; Freitag et al., 2006; Ward et al., 2007; Mosier and Francis, 2008; Sahan and Muyzer, 2008; Jin et al., 2011; Wankel et al., 2011). Their mutual distribution is strongly influenced by salinity, with the *Nitrosomonas* lineages more at the land side and the *Nitrosospira* lineages more at the sea side of the estuaries. However, as already concluded by Francis et al. (2003), no single physical or chemical parameter entirely explains the pattern of diversity along the estuary, suggesting that a complex combination of environmental factors (e.g., oxygen, temperature, nitrate, and ammonium concentrations) may shape the overall level of AOB diversity in a dynamic environment. In our paper, we have indeed shown that factors such as ammonium availability and redox conditions play indeed a role in structuring communities of β-AOB as well. The ecological consequences of a change in community composition of the β-AOB require further study. A change in community composition of β-AOB in the Scheldt estuary due to increasing salinity (De Bie et al., 2001) had in fact no effect on the rate of ammonia oxidation as this was only determined by the availability of ammonium and oxygen, which were both limiting microbial activity. However, a replacement of lineages of β-AOB was essential for the maintenance of the ecological function of ammonia oxidation at changing environmental conditions. The same phenomenon may apply for the studied impounded Black mangrove soils.

### Conflict of interest statement

The authors declare that the research was conducted in the absence of any commercial or financial relationships that could be construed as a potential conflict of interest.

## References

[B1] Bae H. Song J. (2011). GenBank.

[B2] Bernhard A. E. Donn T. Giblin A. E. Stahl D. A. (2005). Loss of diversity of ammonia-oxidizing bacteria correlates with increasing salinity in an estuary system. Environ. Microbiol. 7, 1289–1297 10.1111/j.1462-2920.2005.00808.x 16104852

[B3] Bernhard A. E. Tucker J. Giblin A. E. Stahl D. A. (2007). Functionally distinct communities of ammonia-oxidizing bacteria along an estuarine salinity gradient. Environ. Microbiol. 9, 1439–1447 10.1111/j.1462-2920.2007.01260.x 17504481

[B4] Clark M. W. McConchie D. Lewis D. W. Saenger P. (1998). Redox stratification and heavy metal partitioning in Avicennia-dominated mangrove sediments: a geochemical model. Chem. Geol. 149, 147–171 10.1016/S0009-2541(98)00034-5

[B5] Coci M. Riechmann D. Bodelier P. L. E. Stefani S. Zwart G. Laanbroek H. J. (2005). Effect of salinity on temporal and spatial dynamics of ammonia-oxidising bacteria from intertidal freshwater sediment. FEMS Microbiol. Ecol. 53, 359–368 10.1016/j.femsec.2005.01.016 16329955

[B6] Daebeler A. Abell G. C. Bodelier P. L. E. Bodrossy L. Frampton D. M. Hefting M. M. (2012). Archaeal dominated ammonia-oxidizing communities in Icelandic grassland soils are moderately affected by long-term N fertilization and geothermal heating. Front. Terrestrial Microbiol. 3:14 10.3389/fmicb.2012.00352 23060870 PMC3463987

[B7] de Bie M. J. M. Speksnijder A. G. C. L. Kowalchuk G. A. Schuurman T. Zwart G. Stephen J. R. (2001). Shifts in the dominant populations of ammonia-oxidizing beta-subclass Proteobacteria along the eutrophic Schelde estuary. Aquat. Microb. Ecol. 23, 225–236 10.3354/ame023225

[B8] Dorador C. Busekow A. Vila I. Imhoff J. F. Witzel K. P. (2008). Molecular analysis of enrichment cultures of ammonia oxidizers from the Salar de Huasco, a high altitude saline wetland in northern Chile. Extremophiles 12, 405–414 10.1007/s00792-008-0146-x 18305895 PMC2757604

[B9] Francis C. A. O'Mullan G. D. Ward B. B. (2003). Diversity of ammonia monooxygenase (*amoA*) genes across environmental gradients in Chesapeake Bay sediments. Geobiology 1, 129–140 10.1046/j.1472-4669.2003.00010.x

[B10] Francis C. A. Roberts K. J. Beman J. M. Santoro A. E. Oakley B. B. (2005). Ubiquity and diversity of ammonia-oxidizing archaea in water columns and sediments of the ocean. Proc. Natl. Acad. Sci. U.S.A. 102, 14683–14688 10.1073/pnas.0506625102 16186488 PMC1253578

[B11] Freitag T. E. Chang L. Prosser J. I. (2006). Changes in the community structure and activity of betaproteobacterial ammonia-oxidizing sediment bacteria along a freshwater-marine gradient. Environ. Microbiol. 8, 684–696 10.1111/j.1462-2920.2005.00947.x 16584480

[B12] Jin T. Zhang T. Ye L. Lee O. O. Wong Y. H. Qian P. Y. (2011). Diversity and quantity of ammonia-oxidizing Archaea and Bacteria in sediment of the Pearl River Estuary, China. Appl. Microbiol. Biotechnol. 90, 1137–1145 10.1007/s00253-011-3107-8 21286709 PMC3076564

[B13] Kowalchuk G. A. Stephen J. R. (2001). Ammonia-oxidizing bacteria: a model for molecular microbial ecology. Annu. Rev. Microbiol. 55, 485–529 10.1146/annurev.micro.55.1.485 11544365

[B14] Kowalchuk G. A. Stephen J. R. De Boer W. Prosser J. I. Embley T. M. Woldendorp J. W. (1997). Analysis of ammonia-oxidizing bacteria of the beta subdivision of the class proteobacteria in coastal sand dunes by denaturing gradient gel electrophoresis and sequencing of PCR-amplified 16s ribosomal DNA fragments. Appl. Environ. Microbiol. 63, 1489–1497 9097446 10.1128/aem.63.4.1489-1497.1997 PMC168443

[B15] Kristensen E. King G. M. Holmer M. Banta G. T. Jensen M. H. Hansen K. (1994). Sulfate reduction, acetate turnover and carbon metabolism in sediments of the Ao Nam Bor mangrove, Phuket, Thailand. Mar. Ecol. Prog. Ser. 109, 245–255 10.3354/meps109245

[B16] Laanbroek H. J. Keijzer R. M. Verhoeven J. T. A. Whigham D. F. (2012). The distribution of ammonia-oxidizing betaproteobacteria in stands of Black mangroves (*Avicennia germinans*). Front. Microbiol. 3:153 10.3389/fmicb.2012.00153 22536201 PMC3333478

[B17] Laanbroek H. J. Speksnijder A. G. C. L. (2008). Niche separation of ammonia-oxidizing bacteria across a tidal freshwater marsh. Environ. Microbiol. 10, 3017–3025 10.1111/j.1462-2920.2008.01655.x 18479444

[B18] Li M. Cao H. L. Hong Y. G. Gu J. D. (2011). Spatial distribution and abundances of ammonia-oxidizing archaea (AOA) and ammonia-oxidizing bacteria (AOB) in mangrove sediments. Appl. Microbiol. Biotechnol. 89, 1243–1254 10.1007/s00253-010-2929-0 20953601 PMC3035804

[B19] Li M. Gu J.-D. (2013). Advances in methods for detection of anaerobic ammonium oxidizing (anammox) bacteria. Appl. Microbiol. Biotechnol. 90, 1241–1252 10.1007/s00253-012-4683-y 21476137 PMC3082692

[B20] Ma Y. Wang L. (2007). GenBank.

[B21] Magalhaes C. Bano N. Wiebe W. J. Hollibaugh J. T. Bordalo A. A. (2007). Composition and activity of beta-Proteobacteria ammonia-oxidizing communities associated with intertidal rocky biofilms and sediments of the Douro River estuary, Portugal. J. Appl. Microbiol. 103, 1239–1250 10.1111/j.1365-2672.2007.03390.x 17897228

[B22] McCaig A. E. Embley T. M. Prosser I. J. (1994). Molecular analysis of enrichment cultures of marine ammonia oxidisers. FEMS Microbiol. Lett. 120, 363–367 10.1111/j.1574-6968.1994.tb07059.x 8076810

[B23] Meyer B. Kuever J. (2008). Phylogenetic diversity and spatial distribution of the microbial community associated with the Caribbean deep-water sponge Polymastia cf. corticata by 16rRNA, S, aprA, and amoA gene analysis. Microb. Ecol. 56, 306–321 10.1007/s00248-007-9348-5 18193317 PMC2755779

[B24] Mosier A. C. Francis C. A. (2008). Relative abundance and diversity of ammonia-oxidizing archaea and bacteria in the San Francisco Bay estuary. Environ. Microbiol. 10, 3002–3016 10.1111/j.1462-2920.2008.01764.x 18973621

[B25] Rotthauwe J. H. Witzel K. P. Liesack W. (1997). The ammonia monooxygenase structural gene amoa as a functional marker: molecular fine-scale analysis of natural ammonia-oxidizing populations. Appl. Environ. Microbiol. 63, 4704–4712 9406389 10.1128/aem.63.12.4704-4712.1997 PMC168793

[B26] Sahan E. Muyzer G. (2008). Diversity and spatio-temporal distribution of ammonia-oxidizing Archaea and Bacteria in sediments of the Westerschelde estuary. FEMS Microbiol. Ecol. 64, 175–186 10.1111/j.1574-6941.2008.00462.x 18336555

[B27] Schleper C. Nicol G. W. (2010). Ammonia-oxidising Archaea - physiology, ecology and evolution. Adv. Microb. Physiol. 57:14 10.1016/B978-0-12-381045-8.00001-1 21078440

[B28] Schloss P. D. Westcott S. L. Ryabin T. Hall J. R. Hartmann M. Hollister E. (2009). Introducing mothur: open-source, platform-independent, community-supported software for describing and comparing microbial communities. Appl. Environ. Microbiol. 75, 7537–7541 10.1128/AEM.01541-09 19801464 PMC2786419

[B29] Urakawa H. Kurata S. Fujiwara T. Kuroiwa D. Maki H. Kawabata S. (2006). Characterization and quantification of ammonia-oxidizing bacteria in eutrophic coastal marine sediments using polyphasic molecular approaches and immunofluorescence staining. Environ. Microbiol. 8, 787–803 10.1111/j.1462-2920.2005.00962.x 16623737

[B30] Wankel S. D. Mosier A. C. Hansel C. M. Paytan A. Francis C. A. (2011). Spatial variability in nitrification rates and ammonia-oxidizing microbial communities in the agriculturally impacted elkhorn slough estuary, California. Appl. Environ. Microbiol. 77, 269–280 10.1128/AEM.01318-10 21057023 PMC3019697

[B31] Ward B. B. Eveillard D. Kirshtein J. D. Nelson J. D. Voytek M. A. Jackson G. A. (2007). Ammonia-oxidizing bacterial community composition in estuarine and oceanic environments assessed using a functional gene microarray. Environ. Microbiol. 9, 2522–2538 10.1111/j.1462-2920.2007.01371.x 17803777

[B32] Wickramasinghe S. Borin M. Kotagama S. W. Cochard R. Anceno A. J. Shipin O. V. (2009). Multi-functional pollution mitigation in a rehabilitated mangrove conservation area. Ecol. Eng. 35, 898–907 10.1016/j.ecoleng.2008.12.021

[B33] Zhou J. Z. Bruns M. A. Tiedje J. M. (1996). DNA recovery from soils of diverse composition. Appl. Environ. Microbiol. 62, 316–322 8593035 10.1128/aem.62.2.316-322.1996 PMC167800

