# Supplementary material for: Changes in community composition of ammonia-oxidizing betaproteobacteria from stands of Black mangrove (Avicennia germinans) in response to ammonia enrichment and more oxic conditions
Source: Front Microbiol. 2013 Nov 20;4:343. doi: 10.3389/fmicb.2013.00343 (PMC3834342; doi:10.3389/fmicb.2013.00343)
Supplement: Supplementary file 1 [file Presentation1.PDF]

Supplementary Table 1: Sequence distribution of ammonia-oxidizing bacteria before and after 6 days of incubation in soil slurries that had not been shaken between day 0 and 6. Sequences originated from different vegetation cover types of Black mangrove. Operational Taxonomic Units (OTUs) are based on the partial 16S rRNA gene (440 bp).

Vegetation cover	Dwarf		Sparse		Dense		All	
Sampling day	0	6	0	6	0	6	0	6
OTU01	91	65	18	49	39	39	148	153
OTU02	1	0	43	15	25	4	69	19
OTU03	4	2	23	7	14	9	41	18
OTU04	7	9	26	9	1	5	34	23
OTU05	11	0	0	0	3	0	14	0
OTU06	7	6	0	0	2	0	9	6
OTU07	0	0	1	0	0	0	1	0
OTU08	2	0	1	2	1	0	4	2

Supplementary Table 2: Sequence distribution of ammonia-oxidizing bacteria before and after 6 days of incubation in soil slurries that had been shaken between day 0 and 6. Sequences originated from different vegetation cover types of Black mangrove. Operational Taxonomic Units (OTUs) are based on the partial 16S rRNA gene (440 bp).

Vegetation cover	Dwarf		Sparse		Dense		All	
Sampling day	0	6	0	6	0	6	0	6
OTU01	26	68	26	57	6	15	58	140
OTU02	4	0	35	0	12	2	51	2
OTU03	13	9	11	23	50	22	74	54
OTU04	12	23	6	17	6	9	24	49
OTU05	0	0	0	0	0	0	0	0
OTU06	0	0	2	0	0	0	2	0
OTU07	0	0	0	2	9	14	9	16
OTU08	1	0	1	0	0	0	2	0

Supplementary Table 3A: Spearman Rank Order correlations between archaeal and bacterial amoA gene copy numbers (AOA and AOB, respectively), their mutual ratio and a number of soil characteristics

	log AOA	log AOB	log (AOA/AOB)	moisture content	total soil N	total soil P	extractable ammonium	extractable nitrate	AMP[1]	PAA[2]
log AOA	1.000	0.545	-0.118	0.149	-0.003	0.487	-0.073	-0.365	-0.060	-0.231
log AOB	0.545	1.000	-0.844	0.252	0.212	0.292	-0.266	-0.166	0.054	-0.108
log (AOA/AOB)	-0.118	-0.844	1.000	-0.263	-0.278	-0.012	0.323	0.012	-0.123	0.084
Moisture content	0.149	0.252	-0.263	1.000	0.906	0.107	0.247	0.064	0.642	0.104
total soil N	-0.003	0.212	-0.278	0.906	1.000	-0.054	0.384	0.285	0.797	0.206
total soil P	0.487	0.292	-0.012	0.107	-0.054	1.000	-0.055	-0.481	-0.130	-0.164
extractable ammonium	-0.073	-0.266	0.323	0.247	0.384	-0.055	1.000	0.532	0.450	0.090
extractable nitrate	-0.365	-0.166	0.012	0.064	0.285	-0.481	0.532	1.000	0.430	0.080
AMP[1]	-0.060	0.054	-0.123	0.642	0.797	-0.130	0.450	0.430	1.000	0.249
PAA[2]	-0.231	-0.108	0.084	0.104	0.206	-0.164	0.090	0.080	0.249	1.000
Salinity	-0.355	-0.187	0.080	-0.422	-0.240	-0.403	0.259	0.682	0.073	-0.071
pH	-0.324	-0.332	0.245	-0.557	-0.376	-0.273	0.180	0.461	-0.147	-0.285
NH_4^+	0.014	-0.253	0.303	-0.098	-0.048	0.028	0.282	0.013	0.054	0.171
NO_3^-	-0.443	-0.355	0.230	-0.463	-0.314	-0.389	0.279	0.550	-0.077	0.004
PO_4^{3-}	0.036	-0.098	0.146	0.132	0.083	0.268	0.182	-0.247	-0.028	-0.024
Fe	-0.360	-0.262	0.161	-0.133	0.072	-0.362	0.461	0.731	0.169	-0.010
Ca^{2+}	-0.114	-0.058	-0.073	-0.294	-0.152	-0.200	0.099	0.503	-0.017	-0.135
Mg^{2+}	-0.324	-0.165	0.076	-0.298	-0.159	-0.298	0.275	0.558	0.105	0.051
SO_4^{2-}	-0.388	-0.166	0.007	-0.302	-0.122	-0.409	0.178	0.622	0.089	0.083
Na^+	-0.334	-0.196	0.097	-0.446	-0.236	-0.406	0.291	0.688	0.072	0.005
K^+	-0.266	-0.069	-0.020	-0.405	-0.185	-0.429	0.237	0.697	0.007	0.081

[1] AMP = Anaerobic Mineralization Potential or total amount of degradable organic nitrogen; [2] PAA = Potential Ammonia-oxidizing Activity. Significant ($p<0.05$) correlation are shown in red.

Supplementary Table 3B: Spearman Rank Order correlations between archaeal and bacterial amoA gene copy numbers (AOA and AOB, respectively), their mutual ratio and a selection of pore water characteristics

	Salinity	pH	NH_4^+	NO_3^-	PO_4^{3-}	Fe	Ca^{2+}	Mg^{2+}	SO_4^{2-}	Na^+	K^+
log AOA	-0.355	-0.324	0.014	-0.443	0.036	-0.360	-0.114	-0.324	-0.388	-0.334	-0.266
log AOB	-0.187	-0.332	-0.253	-0.355	-0.098	-0.262	-0.058	-0.165	-0.166	-0.196	-0.069
log (AOA/AOB)	0.080	0.245	0.303	0.230	0.146	0.161	-0.073	0.076	0.007	0.097	-0.020
Moisture content	-0.422	-0.557	-0.098	-0.463	0.132	-0.133	-0.294	-0.298	-0.302	-0.446	-0.405
total soil N	-0.240	-0.376	-0.048	-0.314	0.083	0.072	-0.152	-0.159	-0.122	-0.236	-0.185
total soil P	-0.403	-0.273	0.028	-0.389	0.268	-0.362	-0.200	-0.298	-0.409	-0.406	-0.429
extractable ammonium	0.259	0.180	0.282	0.279	0.182	0.461	0.099	0.275	0.178	0.291	0.237
extractable nitrate	0.682	0.461	0.013	0.550	-0.247	0.731	0.503	0.558	0.622	0.688	0.697
AMP[1]	0.073	-0.147	0.054	-0.077	-0.028	0.169	-0.017	0.105	0.089	0.072	0.007
PAA[2]	-0.071	-0.285	0.171	0.004	-0.024	-0.010	-0.135	0.051	0.083	0.005	0.081
Salinity	1.000	0.539	-0.082	0.769	-0.343	0.585	0.640	0.867	0.859	0.943	0.822
pH	0.539	1.000	0.142	0.584	-0.078	0.422	0.444	0.369	0.375	0.553	0.540
NH_4^+	-0.082	0.142	1.000	0.142	0.431	0.078	-0.027	-0.010	-0.079	0.011	0.062
NO_{3-}	0.769	0.584	0.142	1.000	-0.190	0.539	0.436	0.665	0.678	0.761	0.651
PO_4^{3-}	-0.343	-0.078	0.431	-0.190	1.000	-0.063	-0.412	-0.263	-0.370	-0.317	-0.214
Fe	0.585	0.422	0.078	0.539	-0.063	1.000	0.361	0.499	0.555	0.624	0.617
Ca^{2+}	0.640	0.444	-0.027	0.436	-0.412	0.361	1.000	0.708	0.731	0.711	0.703
Mg^{2+}	0.867	0.369	-0.010	0.665	-0.263	0.499	0.708	1.000	0.910	0.928	0.773
SO_4^{2-}	0.859	0.375	-0.079	0.678	-0.370	0.555	0.731	0.910	1.000	0.914	0.809
Na^+	0.943	0.553	0.011	0.761	-0.317	0.624	0.711	0.928	0.914	1.000	0.880
K^+	0.822	0.540	0.062	0.651	-0.214	0.617	0.703	0.773	0.809	0.880	1.000

Significant ($p<0.05$) correlation are shown in red.
